# Validity Study of the R-PLA, a Resilience Scale for People Living with HIV

**DOI:** 10.16966/2380-5536.170

**Published:** 2019-12-20

**Authors:** Jinxiang Hu, Julianne M Serovich, Monique J Brown, Judy A Kimberly, Yi-Hsin Chen

**Affiliations:** 1 Department of Biostatistics and Data Science, University of Kansas Medical Center, USA; 2 College of Behavioral and Community Science, University of South Florida, USA; 3 Department of Epidemiology and Biostatistics, Arnold School of Public Health, University of South Carolina, USA; 4 Brown University, USA; 5 Department of Educational and Psychological Studies, College of Education, University of South Florida, USA

**Keywords:** HIV, Resilience, Rasch model, Validity

## Abstract

This study provides psychometric assessment of a resilience scale with a sample of women living with HIV. Baseline data were used from a longitudinal HIV disclosure study of 124 women aged between 18–63 collected between 2001 and 2004 in a large Midwestern city. The Rasch model was used to examine the psychometric properties of the resilience scale. Results indicated that the resilience instrument meets the Rasch model application assumptions. Evidence of validity suggested the resilience instrument demonstrated good item and person fit, as well as good item and person reliability. Most items showed measurement invariance across different age and racial groups. The findings suggest that the resilience scale is suitable for use in the measurement of resilience among women living with HIV.

## Introduction

Resilience is a dimension of mental health [1,2], and can be defined as a process or a personality trait [3,4]. From a process perspective, resilience can be defined as a dynamic process interacting with stress and adversity [1,4–7]. From a personality trait perspective, resilience has been defined as an individual characteristic that dampens the negative effects of stress and facilitates adaptation [1,5].

Resilience has been positively related to mental health [4,8–13] and quality of life [14,15]. For example, Schure MB, et al. [8] found that higher levels of resilience were associated with higher levels of mental and physical health among older American Indians. Resilience has also been positively associated with health-related quality of life among people 50 years or older living with HIV/AIDS [15]. However, the use of resilience as an outcome has been criticized for ambiguity as a construct and lack of validated measures [5,16,17]. Therefore, resilience as an outcome is not often used in research [1,4,18].

To the best of our knowledge, resilience has not been studied extensively in the context of the lives of women living with HIV. In 2016, approximately 19% of the newly diagnosed HIV patients in the U.S. were women [19]. Women experience gender inequalities in many aspects of life, such as employment, income, and education [3]. Despite these disadvantages, women tend to demonstrate a higher prevalence of resilience than men [20].

For HIV researchers seeking a better understanding of resilience so as to improve the lives of women living with HIV, a psychometrically sound measure is important. Numerous measures of resilience are currently available, and each has been developed for specific purposes or populations. For example, scales have been developed for measuring resilience in the general population, such as the Connor-Davidson Resilience Scale (CD-RISC) [21,22], the Resilience Scale (RS) [23], and the Brief Resilience Scale (BRS) [24]. Others have been more specific and geared toward certain populations, such as adults (e.g., Resilience Scale for Adults [RSA]) [25] and adolescents (e.g., Adolescent Resilience Scale [ARS]) [26]. A review study showed that as many instruments of different lengths exist to measure resilience among different populations, it is difficult to compare resilience across different studies, and no consensus exists about dimensionality [17]. For example, the CD-RISC has five dimensions (Personal competence, Trust, Positive Acceptance of Change, Control, and Spiritual Influence) and 25 items. The RS has two dimensions (Personal Competence and Acceptance of Self and Life) and 25 items. The BRS [24] has one dimension of resilience and 6 questions. In order to decrease administration time but still obtain psychometric stability of the CD-RISC, shorter versions of CD-RISC10 [27], CD-RISC2 [28] with 1 dimension were proposed and have been used in other studies [21,29–34]. In addition, scales have been designed to be used with various medical conditions, such as chronic diseases of asthma or diabetes [35]. Researchers have been using scales such as CD-RISC to measure resilience of people living with HIV [3,11]. A resilience scale designed for those living with HIV has yet to be developed to fill this gap.

A search of the literature suggested that Mosack KE [36] has been the only researcher who has attempted to quantify the measure of resilience in people living with HIV/AIDS (R-PLA). The R-PLA scale consists of 28 items and used classical test theory, which is based on observed scores. However, the original scale was not validated on populations other than those in the original study to test its psychometric stability. For the scale to be of better use to future researchers, while demonstrating sound psychometric qualities, we propose a validated shortened version of the R-PLA.

After expert (coauthors of this paper; details in content validity section) review, and with theoretical guidance from Kobasa (1979), Rutter M [7], and Connor KM and Davidson JR [22] only 12 items were retained for analysis in the current study (details in the content validity section). The current study used Item Response Theory (IRT) [37] to validate the R-PLA scale. The IRT is a modern test theory that assumes a continuous latent score (resilience in this study) in all participants, in comparison to Classical Test Theory (CTT) that uses observed scores. For the original study, Mosack KE [36] used CTT to measure resilience score of the patients. However, a key limitation to CTT is that the observed scores of the participants are dependent on the test [38]. For example, if the questions are easy to agree upon, participants’ average resilience scores can be inflated; if the questions are too hard to agree upon, participants’ average resilience scores can be reduced. Therefore, for the scale to be of better use to future researchers, a validation of the R-PLA measure that overcomes this test-dependent disadvantage is necessary. The purpose of the present study was to provide the validity evidence for the R-PLA scale using IRT with a sample of women living with HIV.

## Methods

### Participants

Data for this study were drawn from the baseline assessment of a longitudinal HIV disclosure study of women, which was conducted between 2001 and 2004 in a large Midwestern city. Participants were recruited through local organizations and institutions that served women living with HIV, including HIV/AIDS service organizations, a children’s hospital, and a clinical trial unit (ACTU) associated with a large university. Flyers were posted in waiting areas, and the study was advertised in newsletters of the HIV/AIDS service organizations. At the children’s hospital and ACTU, flyers were posted in waiting rooms, and medical staff and attending physicians approached and informed potential participants about the study. To be eligible for the study, women had to be 18 years of age or older and living with HIV. Due to the variety of recruitment strategies employed, it was not possible to calculate a participation rate, as medical staff could not track whether those patients they had referred actually enrolled in the study. One hundred twenty-four women were enrolled and provided data for this study. Data were collected *via* paper and pencil questionnaires, and all participants provided written, signed informed consent prior to participation. The study, its methods, and protocols were approved prior to data collection by the Ohio State University Institutional Review Board.

### Measures

The R-PLA is a 12-item measure adapted from Mosack’s measure assessing resilience in people living with HIV (Table 1 near here). The initial testing of this scale demonstrated adequate internal consistency, test-retest reliability and construct validity within a sample of HIV-positive men and women [36]. Responses were Likert-type responses ranging from 1 (strongly disagree) to 5 (strongly agree) with higher scores indicating higher resilience (total score range: 12–60). The five-point Likert scale was recorded as binary with 4 to 5 representing “agree” and 1 to 3 representing “disagree”, considering the small sample size (N=124) and the response curves of the middle category were almost overlapping with the second category for each item (Response curves of the items could be obtained from the first author upon request).

### Statistical analysis

The validation process was completed by using the Rasch model [39] to provide psychometric assessment to the R-PLA. The Rasch model belongs to the IRT family and fits items into one of two categories: 1 for “yes” and 0 for “no”. The Rasch model requires a sample size of 100 or larger [40]. The Rasch model estimates the probability (P) of a person with ability agreeing to an item with difficulty level, and can be represented by Equation 1:

(1) $$P=\frac{\exp(\theta -b)}{1+\exp(\theta -b)}$$

The person’s ability θ in this study refers to the participant’s resilience score, and the item difficulty b stands for a resilience score of 0 having a 50% chance agreeing to an item. If a person’s resilience score was greater than the item difficulty, the person was likely to agree to the item and *vice versa*. The analysis was conducted in the R language and environment [41].

The validity of the resilience scale was addressed *via* the assessment of model assumption, the content validity, model-data fit, the reliability, and the measurement invariance of the resilience scale. The assumptions of the Rasch model were assessed by the unidimensionality test in the ltm package [42] and the inter-item correlation test for local independence in the eRm package [43]. Rasch model-data fit was assessed by χ^2^ statistics [44], item fit mean square and person fit mean square [43]. Mean square between 0.5 and 1.5 were considered good for measurement [45].

Reliability of the resilience scale was investigated in the ltm package and eRm package by examining the person reliability, the item reliability, and the person item map. The reliability in the Rasch model indicated the likelihood that the estimate of person or item by the Rasch model could be reproduced [45]. Person reliability is similar to Cronbach’s alpha [45]. The wider the person ability range is and/or the more items present, the higher the person reliability.

Measurement invariance was crucial in that it assured that the R-PLA measured the latent resilience score accurately regardless of group difference. Measurement invariance was investigated by examining the differential item functioning (DIF) using the likelihood ratio test (LRT; Table 2 near here) in the difR package [46]. DIF in this study assessed whether participants of different groups (age ≥ or <mean age 37, or Caucasian *vs.* minority) responded the same to each item given the same resilience score. We tested DIF across different age and racial/ethnic groups [20,47,48].

## Results

### R-PLA descriptive statistics

The average age of participants (N=124) was 37.77 years (SD=9.43 years; Range 18–63 years) with average time since diagnosis of 6.49 years (SD=4.07; Range=0.08–18.33 years). Participants had, on average, two children (Mean=2.20, SD=1.63, Range=0–5). Twenty-eight percent (28%) of participants reported monthly income over $1,000 (Mean=$817.46, SD=$ 885.93, Range=$0-$6600). The majority of participants were African-American (68.5%; n=85); 25% were Caucasian (n=31), and 3% were Hispanic/Latino (n=4). Over 33% of participants reported their relationship status as single (n=41); 17.7% were dating (n=22); 33.9% were married/partnered (n=42); 10.5% were divorced (n=13), and 4.8% widowed (n=6). Approximately 42.7% of the participants reported having completed some college or above (n=53), and a majority of the participants were unemployed (78.2%; n=97).

The observed mean representing the actual proportion of participants’ agreement (as well as model estimated proportions) on each item were rank-ordered from low to high (Table 1). Item 5 had the lowest proportion of agreement at 0.55 (55% of the participants agreed), and the last item had the highest proportion of agreement at 0.86 (86% of the participants agreed). The Rasch mean represented the model estimated portion of agreement from the participants. The model estimated means were either the same or very close to the observed means, indicating a good model-data fit.

### Unidimensionality and local independence assumptions

**Figure 1:**
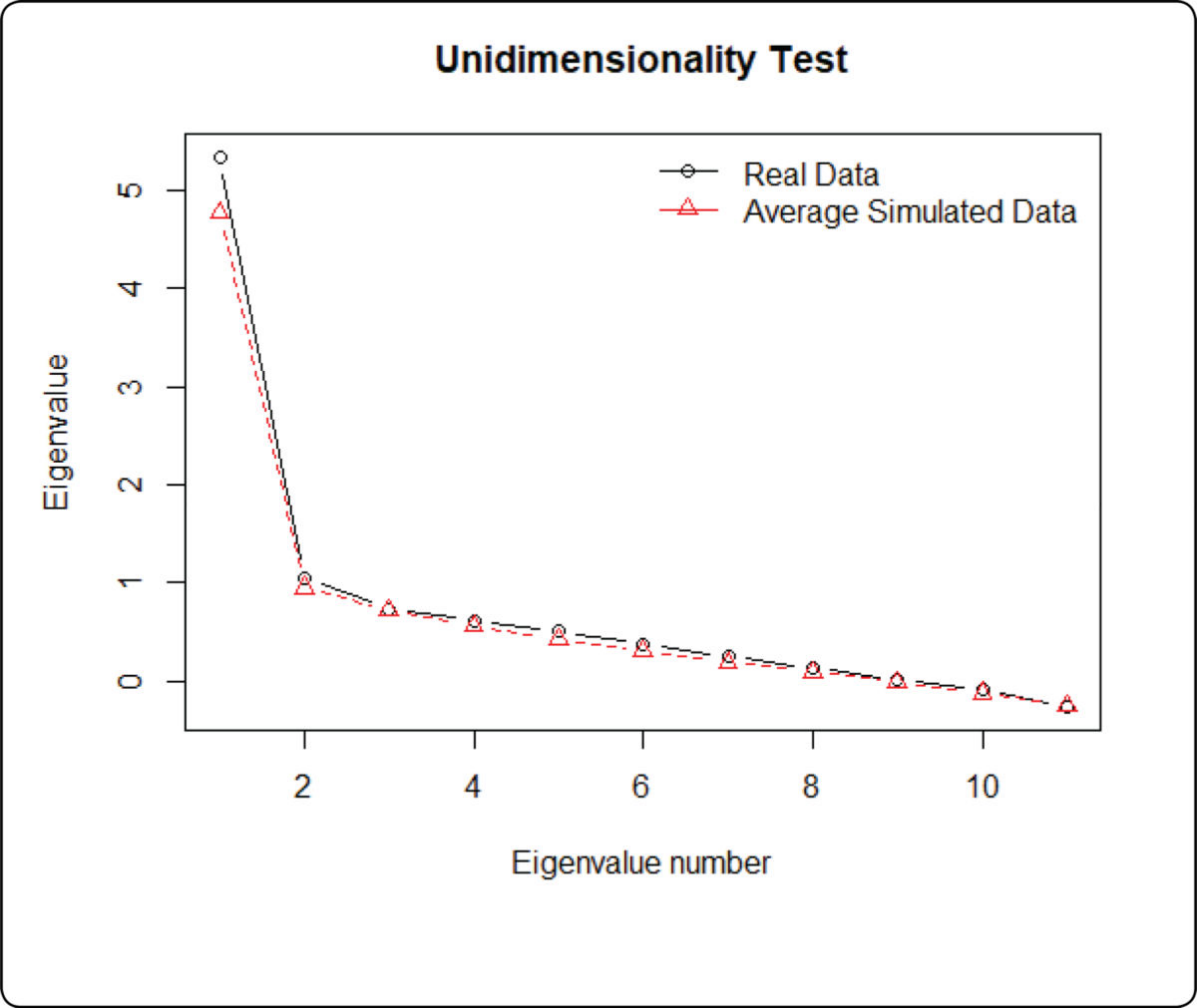
Unidimensionality test.

The unidimensionality assumption requires that all the items measure only one latent trait from the participants, which is the resilience score in this study. The local independence assumption states that all the items are independent, and the latent trait (i.e., resilience score) is the only factor that correlates the items. The unidimensionality test [49] for binary items implemented in the ltm package was conducted. As shown in figure 1, results indicated that the observed resilience data showed one dimension structure (Figure 1). Local independence was assessed with testing the inter-item correlations of the resilience items [50]. Results showed that only item 10 (I am stronger than HIV and plan to live a long life) and item 12 (I will not let HIV get the best of me) were not independent at alpha=0.01 level (r=0.89, p<0.01). We decided to remove item 10 and shorten the instrument to 11 questions.

### Content validity

Content validity was assessed with expert review and the Rasch item-measure correlations (Table 1). Expert review was conducted by three HIV research experts (coauthors of this paper) with content area expertise in HIV/AIDS research among vulnerable populations, epidemiology and women’s health, and HIV social support. The experts were assisted by the Connor KM and Davidson JR [22] and adapted the items from Mosack KE [36]. Seven items were “HIV” specific, other items were guided by Connor and Davidson scale. Some items corresponded to the “optimism” aspect of resilience (items 2,3,4,5, and 6); two items aligned with the “self-efficacy” aspect (items 8 and 9); two items corresponded to the “view change or stress as challenge or opportunity” aspect (items 7 and 11; and two items aligned with other aspects of resilience (item 10 with the “adaptability” aspect, and item 1 with the “personal goal” aspect).

The Rasch item-measure correlation represented correlation between the responses on each item and the total score excluding that specific item. The item-measure correlation of the resilience scale ranged from 0.31 to 0.60, indicating that all items had positive, moderately high correlations with the total resilience scale score.

### Model fit

**Figure 2:**
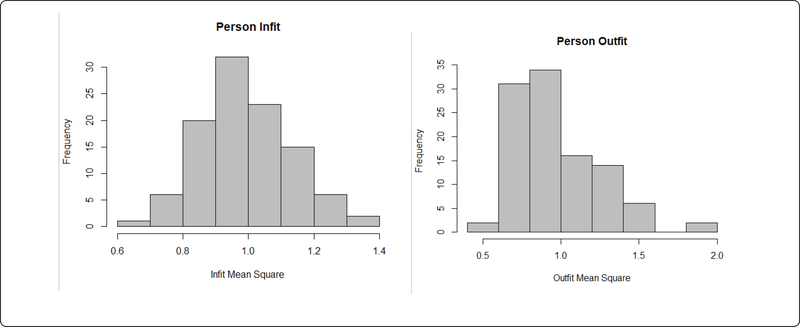
Person Fit Statistics Distribution.

All 11 items fit the Rasch model well (Table 1). Infit values ranged from 0.77 to 1.20. Outfit values were also good except item 11 was slightly under 0.5(0.46). Because the infit statistics are more sensitive to unexpected observations of persons on items that match their ability level [45], infit statistics are more informative when investigating the fit of the items to the Rasch model [51–53]. Therefore, item 11 was kept in the scale. The person fit chi square results showed that all participants fit the model (p>0.05). Person infit mean square were within the range of 0.5 to 1.5. Only three persons showed a misfit in outfit mean square (Figure 2). The findings showed that observed responses of all persons were consistent with the response patterns predicted by the model.

### Reliability

**Figure 3:**
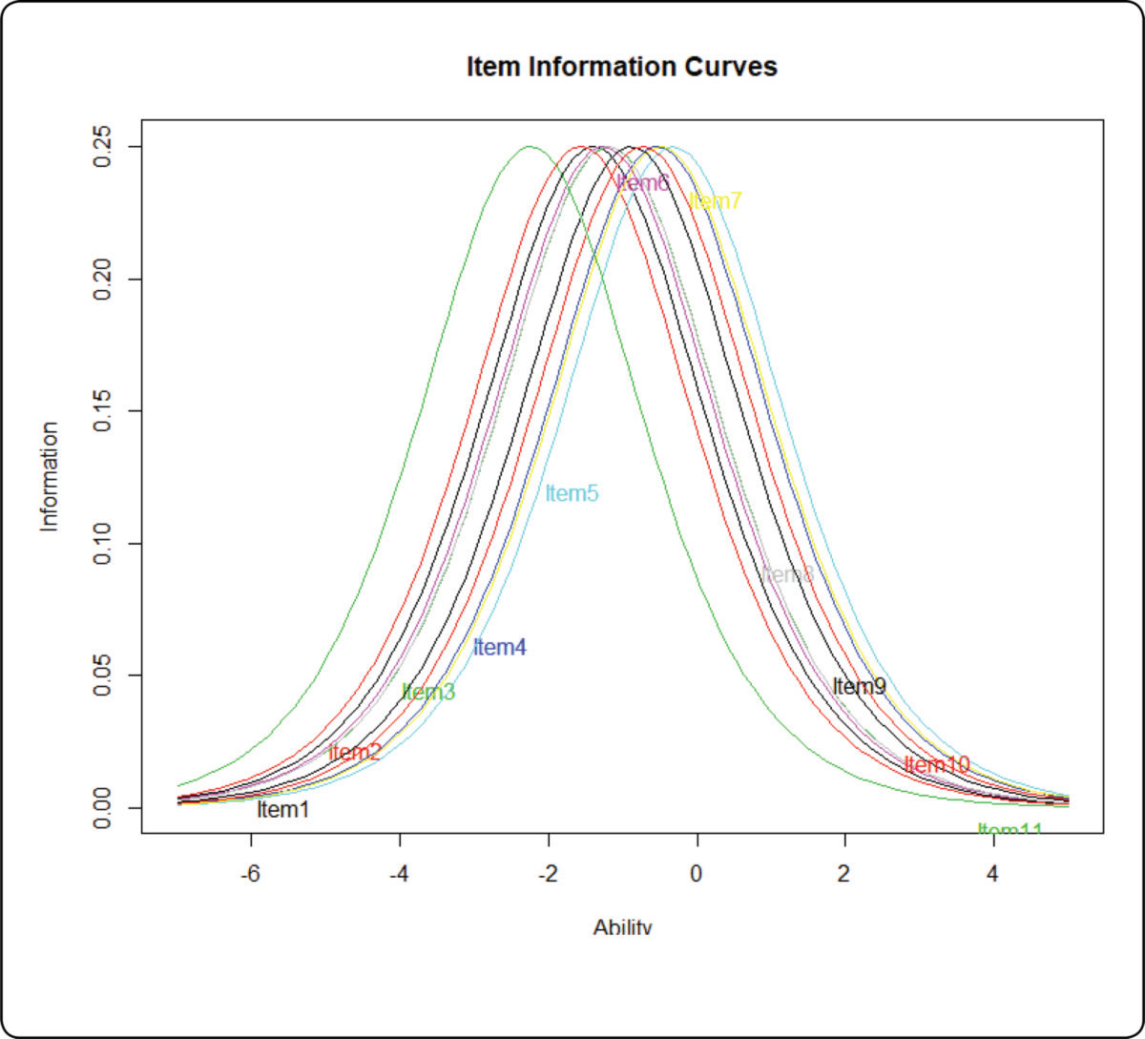
Item Information.

Person reliability of the resilience scale was 0.80. The item reliability was assessed by item information and item difficulty variance. The item provides the most information to the person whose ability level is closer to the item difficulty level. Item information can be visualized by the Item Information Curve (IIC). The item information of the R-PLA spreads evenly along the latent resilience score (Figure 3 near here), indicating wide item information coverage along the latent resilience scale and high reliability. The wider the range of the item difficulty levels, the more overall test information, and the higher the reliability of the whole scale [45]. The item difficulty had a wide range from −2.24 to −0.32 (Table 2). Based on the item information and item difficulty variance, the resilience scale had high item reliability.

**Figure 4:**
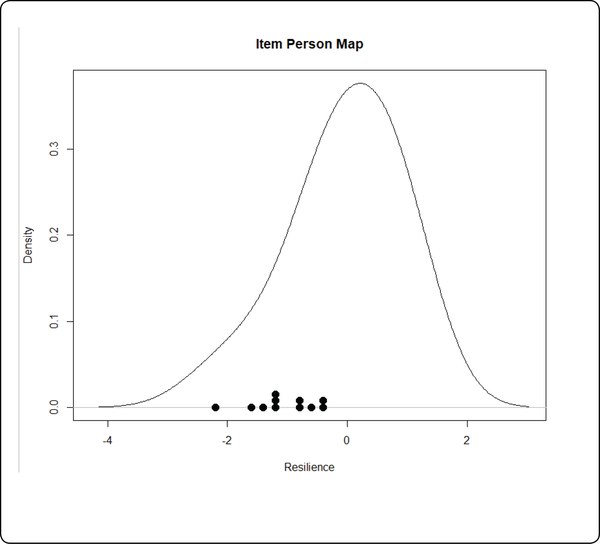
Person-item Map.

The person-item map (Figure 4 near here) provides graphical evidence for reliability. In the person-item map the resilience score of the participants were plotted on the same graph with the R-PLA items to provide a visualization of the matching of people’s ability and the item difficulty. The more matched they are, the higher the reliability of the R-PLA scale. The items were evenly spread out along the resilience scale, indicating that items and persons matched well and that the scale has high reliability.

### DIF

Results indicated that item 4 showed DIF on age at significance level of .01 and favored women who were older than the average age (Table 2). In other words, given the same latent scores of resilience, women who were older (>mean 37) were more likely to believe “there is something good that come from this disease”. Results also showed DIF by different racial/ethnic groups on item 2, item 3, and item 8. Specifically, given the same latent scores of resilience, Caucasian women were more likely to agree on “see the positive in people” (item 2) and “when I have a question about HIV, I know where to find the answer” (item 8), whereas women of minority were more likely to agree on “believe things will only get better for me” (item 3).

## Conclusion and Discussion

Resilience is an important factor for the mental health of people living with HIV. To date, the R-PLA is the only measure that assesses resilience specifically in this population. Validity evidence for this scale in other similar samples is important for the scale to be widely adopted by HIV researchers. Based on the results from the current study in which we measured resilience scores among HIV-positive women, all R-PLA items showed good psychometric qualities in terms of item fit, person fit, item reliability, and person reliability.

The DIF test showed women older than 37 years tended to agree more on item “something good has come from living with HIV”. Although findings from the current study suggest that most of the items did not favor people of either age group, this finding raises an interesting question: Does resilience increase with age among people living with HIV? In the general literature, inconsistencies appear with regard to the role of age in resilience. Although some researchers have shown no significant association between age and resilience [23,54]. Lundman B, et al. [47] suggested that such a relationship may exist. Their measure of resilience (the resilience scale; RS) was used in a large sample (N=1,719, out of which 1,248 were women), and results showed that resilience was significantly associated with increasing age. Bonanno GA, et al. [20] also found that people over 65 years of age were more likely to be resilient than people aged 18–24 years. Another study by Rothermund K and Brandtstadter J [48] suggested resilience may decrease after 70 years of age. In the future, researchers might consider examining this issue further among people living with HIV, such as determining whether resilience changes with age and/or whether resilience is related to time since diagnosis.

The DIF test also showed that women of different racial groups responded differently to items 2, 3, and 8. For example, items 2 and 8 favored Caucasian/White women. That is, given the same resilience scores, Caucasian/White women tended to agree more on seeing the positive in people and tended to agree more on knowing where to find an answer when facing questions about HIV. In contrast, Item 3 favored racial minority women, indicating that racial minority women were more likely to agree that things would get better for them than Caucasian/White participants. To date and to our knowledge, very few studies have related race or ethnicity to resilience. Bonanno GA, et al. [20] conducted a study investigating the relationship between race and resilience and found no difference between African Americans and Whites in the prevalence of resilience.

Literature in resilience also suggests that resilience may vary not only at the cultural level, but also at the individual level [1,55]. Thus, items 2 and item 3 favoring different racial groups may have been due to both cultural and individual reasons. Also, an interaction may occur at the individual and cultural level; however, we were unable to test this interaction in the current study. Future researchers may consider conducting research focused on the interaction between individuality and culture, and resilience. Dale SK, et al. [3] found that among HIV-positive women, employment was significantly associated with resilience. The fact that Caucasian/White women in this study had a higher employment rate (26% in the Caucasian/White group *vs*. 19% in the ethnic minority group) may explain why Caucasians/White was more likely to agree on item 2.

It should also be noted that both items 2 and 3 related to the optimism aspect of resilience. A search of the literature showed two studies of race and optimism have been conducted [56]. Both compared optimism scores across different racial groups and concluded that Caucasian/White participants had lower optimism mean scores than other races. Burke KL, et al. [56] showed that Blacks had significant higher optimism score than Whites, and Black women had higher mean optimism scores than White women. These findings may explain why item 3 favored the racial minority group because Blacks accounted for 91.4% of the racial minority group in the current study.

Item 8 reflects the self-efficacy aspect of resilience. Self-efficacy has been an important indicator of resiliency [57], and African Americans had lower levels of perceived self-efficacy for various reasons, such as socioeconomic status, employment, and pay [58]. These self-efficacy related differences may explain the difference why racial minority women in the current study were less likely to agree on item 8.

In summary, the results of this study are consistent with what we know about resilience and its relationship with other relevant variables. The results of the item analysis of the R-PLA implied that this resilience scale is a valid measure of resilience among women living with HIV. Because the DIF items were found across different age and race groups in this study, making comparisons of resilience scores between age and race groups should be cautioned and some adjusted procedures might be taken. For instance, to compare the resilience scores between HIV women over and below 37 years old, the response of item 4 might be removed. For the comparison between Caucasians and minorities, the responses of items 3 and 8 might be kept because of the DIF cancellation effect at the scale level, but item 2 might be removed. To the author’s best knowledge, very few studies have examined DIF by gender on the resilience scales available. Only one paper [59] studied the CD-RISC resilience scale in Australia and found measurement invariance across gender. We would thus expect the results of the R-PLA would hold in men as well.

Limitation of this study includes us only examined the content validity, item fit, person fit, reliability, and DIF across different age and racial groups. In the future, researchers could also consider examining the convergent criteria validity of the R-PLA scale, for example, comparing the results of the R-PLA scale to the CD-RISC or the RS. We proposed one dimension of resilience and a shortened version of the R-PLA, which may not be as comprehensive as the original scale, but shortened self-reported scales reduce time and may have more clinical applicability than longer scales. We dichotomized the categories due to small sample size, which may cause loss of information. Also, participants in the current study were women, which may limit the generalizability of this study to men. In the future, researchers should investigate psychometric properties of the 11-item R-PLA scale with five categories using a larger sample size that includes men. Future research can focus on the validation of the R-PLA scale among other populations living with HIV (such as men who have sex with men and people in other regions of the US and in other countries) and examine the measurement invariance across gender. Additionally, the cross-sectional design prohibited assessment of the temporal relation of resilience and HIV.

## Figures and Tables

**Table 1:** Resilience Descriptive Statistics and Rasch Analysis Results.

Item Ordering	R-PLA Items	Observed Mean	Rasch Mean	Item-measure correlation	χ^2^	*p*	Infit MSQ	Outfit MSQ
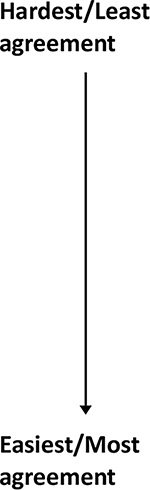	5. will live until they find a cure for HIV	0.55	0.56	0.50	10.65	0.22	0.90	0.81
	7. when faced with a difficult situation, I enjoy working through the problem	0.59	0.59	0.40	4.05	0.85	1.06	1.06
	4. believe there is something good that has come from this disease	0.60	0.60	0.47	4.50	0.80	0.97	0.91
	10. Even though I have HIV, I can do a lot of the same things I did before I got this disease	0.64	0.64	0.34	10.81	0.21	1.19	1.30
	9. believe that I am in control of my health	0.67	0.67	0.58	9.71	0.28	0.80	0.72
	3.believe that things will only get better for me	0.71	0.72	0.50	13.20	0.11	0.91	0.81
	6.am more than HIV-positive	0.72	0.73	0.31	3.43	0.90	1.20	1.52
	1.have a good idea of the things I want to accomplish in my life	0.74	0.75	0.47	8.50	0.38	0.95	1.02
	8. when I have a question about HIV, I know where I can find the answer	0.75	0.72	0.56	9.52	0.29	0.88	0.73
	2.see the positive in people	0.77	0.78	0.32	4.27	0.83	1.18	1.32
	11. Will not let HIV get the best of me	0.86	0.86	0.60	8.99	0.34	0.77	0.46

**Note:** MSQ=Mean Square

**Table 2:** Item Difficulty and DIF Results across Different Age and Race Groups.

Item Ordering	R-PLA Items	*b*	*b_se_*	DIF on age		DIF on race	
				b<mean age	b>mean age	b Caucasian (24.8%)	b Minority (68%)
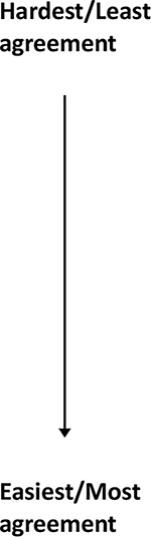	5. will live until they find a cure for HIV	−0.32	0.17	−0.44	−0.21	0.52	−0.60
	7. when faced with a difficult situation, I enjoy working through the problem	−0.48	0.17	−0.52	−0.41	0.01	−0.65
	4. believe there is something good that has come from this disease	−0.53	0.17	−0.10	−0.92*	−0.07	−0.69
	10. Even though I have HIV, I can do a lot of the same things I did before I got this disease	−0.72	0.17	−0.98	−0.49	−0.73	−0.72
	9. believe that I am in control of my health	−0.88	0.20	−1.29	−0.54	−0.56	−1.00
	3.believe that things will only get better for me	−1.19	0.19	−1.39	−1.01	−0.07	−1.67*
	6.am more than HIV-positive	−1.25	0.19	−1.33	−1.19	−1.50	−1.17
	1.have a good idea of the things I want to accomplish in my life	−1.39	0.20	−1.61	−1.21	−1.50	−1.35
	8. when I have a question about HIV, I know where I can find the answer	−1.19	0.19	−1.29	−1.11	−1.99*	−0.97
	2.see the positive in people	−1.56	0.21	−1.89	−1.30	−2.67*	−1.29
	11. Will not let HIV get the best of me	−2.24	0.14	−2.37	−2.12	−1.73	−2.45

**Note:** *b*=item difficulty; *b_se_*=Standard error of item difficulty; DIF=Differential Item Functioning

* indicates DIF *p*<0.01
